# The “Vessel through Strait” Sign is a Signature Radiological Sign for the Diagnosis of Left Hepatic Artery Variation

**DOI:** 10.1038/srep23922

**Published:** 2016-04-04

**Authors:** Guanghua Rong, Zhijun Wang, Ximing Wang, Qiang Yu, Lin Zhou, Huaming Wang, Junhua Zhang, Jinghui Dong, Wei Ma, Weimin An, Hui Ren, Zhen Zeng, Yinying Lu, Yongwu Li

**Affiliations:** 1 Comprehensive Liver Cancer Center, the 302 Hospital, 100 Xi-Si-Huan Middle Road, Beijing 100039, China; 2 Department of Interventional Radiology, the General Hospital of PLA, Beijing 100853, China; 3 Department of Interventional Radiology, the 302 Hospital, 100 Xi-Si-Huan Middle Road, Beijing 100039, China; 4 Department of Radiotherapy, the 302 Hospital, 100 Xi-Si-Huan Middle Road, Beijing 100039, China; 5 Department of Radiology, the 302 Hospital, 100 Xi-Si-Huan Middle Road, Beijing 100039, China; 6 Department of Liver Transplantation, the 302 Hospital, 100 Xi-Si-Huan Middle Road, Beijing 100039, China

## Abstract

An aberrant artery (AA) can frequently be observed coursing through the fissure for the ligamentum venosum (FLV) which was termed the “vessel through strait” sign (VTSS) by us. Fundamental data including the incidence, anatomical composition and clinical significance of VTSS and the AAs composing VTSS are still lacking. We sought to give a systematic demonstration on this issue in the present study. VTSS was respectively analyzed in 2,275 patients and was observed in 357 of them. Interestingly, 319 (89.4%) out of the 357 patients exhibiting VTSS were proved to have left hepatic artery variation (LHAV) (247 with replaced left hepatic artery, 64 with accessory left hepatic artery and 8 with variant common hepatic artery). We therefore hypothesized that VTSS could be a sign that strongly associated with LHAV and could be used for its diagnosis. In the following validating analysis, VTSS gained a sensitivity of 96.3% and a specificity of 98.3% for the diagnosis of LHAV in another bicenter cohort consisted of 1,329 patients. In conclusion, VTSS is a signature radiological sign of LHAV which could be used as an easy and specific method for the diagnosis of LHAV.

An aberrant artery (AA) can be frequently observed coursing through the fissure for the ligamentum venosum (FLV) on axial contrast-enhanced computed tomography (CT) or magnetic resonance imaging (MRI) scans (Fig. 1-A). We termed this manifestation the “vessel through strait” sign (VTSS), as the AA highly resembles a “vessel” which is sailing though a “strait” composed of segment I (S1) and segment II (S2) of the liver (Fig. 1-B). VTSS is estimated to be observed in approximately 15–20% of the general population according to our experience, however, despite being sparsely described as the variant left hepatic artery (LHA)^1^,^2^, or variant common hepatic artery (CHA)^3^ or accessory left gastric artery (LGA)^4^, current knowledge about VTSS and the AAs composing VTSS is very limit, fundamental data including its incidence, anatomical composition and clinical significance are lacking. To address this issue, in the present study, we respectively analyzed the hepatic arteriographic and CT/MRI data in 2,275 patients receiving transcatheter arterial chemoembolization (TACE) with a particular focusing on the VTSS. A very interesting result of our analysis was that nearly 90% of the patients exhibiting VTSS were proved to have left hepatic artery variation (LHAV). The strong association between VTSS and LHAV naturally drove us to propose and validate the hypothesis that VTSS is a signature radiographic sign of LHAV that could be used for its diagnosis.

LHAV include type II, IV, V, VII, VIII and X of Michel’s classification of hepatic artery variation (HAV)^5^, which occur in approximately 12–22% of the general population and represent the second most common pattern of HAV^5^,^6^,^7^. Preoperative awareness of LHAV is therefore important for the planning and performance of all of the surgical or radiological interventional procedures arranged in the left hepatic lobe. Currently, hepatic artery variations (HAVs) including LHAV are mainly detected by digital subtraction hepatic arteriography (DSHA) or computed tomographic angiography (CTA). However, DSHA is invasive and cannot be used preoperatively, whereas CTA requires an additional reconstruction procedure that might require extra time and expense and is not routinely applied to all patients. Thus, clinical application of VTSS may provide an easy and specific solution for the non-invasive diagnosis of LHAV. For this purpose, we conducted a validating analysis to evaluate the usefulness of VTSS as a diagnostic sign of LHAV in another bicenter series of 1,329 patients.

## Results

### The incidence of VTSS and the anatomical compositions of the AAs seen in VTSS

**Figure 1:**
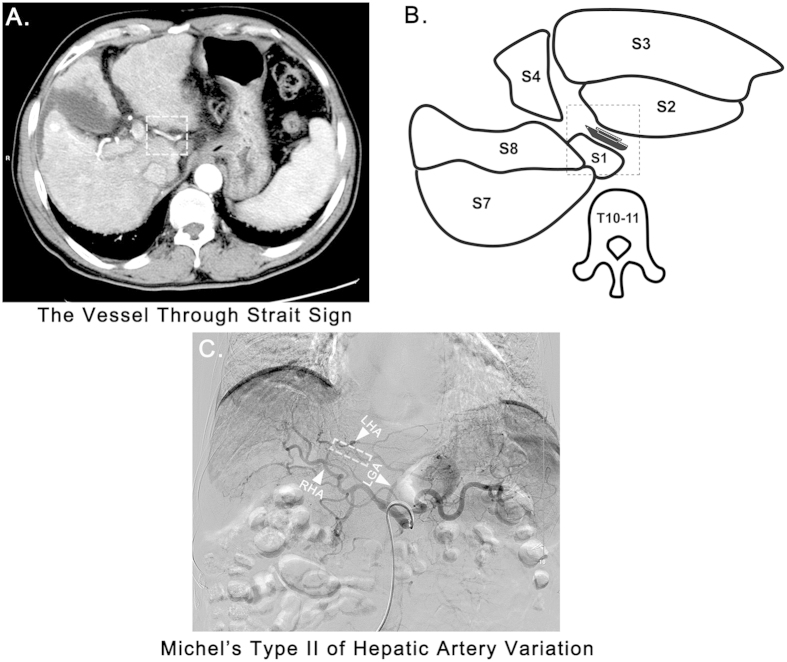
(**A**) A typical VTSS (white dashed square) formed by a replaced LHA entering liver through FLV was seen in the arterial phase of a contrast-enhanced CT scan. (**B**) A schematic diagram of VTSS. The vessel represents the aberrant artery, which is sailing though a “strait” composed of S1 and S2 of the liver (**C**) The patient was confirmed to have a replaced LHA arising from LGA (Michel’s type II HAV) by DSHA. The corresponding part of the replaced LHA forming VTSS was highlighted by the white dashed rectangle. LHA, left hepatic artery; LGA, left gastric artery; RHA, right hepatic artery; S, segment of the liver; T, thoracic vertebrae.

The presence of VTSS was screened in a training cohort of 2,275 patients and was identified in 357 (15.7%) of them. Representative images of VTSS are shown in Fig. 1 and the supplementary figures. In each patient with VTSS, the anatomical property of the observed AA was further analysed according to the hepatic arteriography data. As shown in Table 1, the anatomical composition of the VTSS-associated AA was as follows: replaced LHA (n = 246, 68.9%, Fig. 1-C), accessory LHA (n = 64, 17.9%, supplementary fig. 1), common hepatic artery (CHA) (n = 8, 2.2%, supplementary fig. 2), accessory LGA (n = 26, 7.3%, supplementary fig. 3), left inferior phrenic artery (LIPA) (n = 3, 0.8%, supplementary fig. 4) and the common trunk of accessory LGA and LIPA (n = 10, 2.9%, supplementary fig. 5). Thus, in total, 89.1% (318/357) of the patients with VTSS do indeed have LHAV. We further demonstrated the prevalence of VTSS in all of the 2,275 patients according to their Michel’s classification results. The global profile of HAV in the 2,275 patients and the incidence of VTSS of each Michel’s classification type are shown in Table 2. Overall, LHAV was detected in 318 (14.1%) patients, and VTSS was observed in 312 (98.1%) of them.

### The usefulness of VTSS as a diagnostic sign of LHAV

Our analysis indicated that VTSS was strongly associated with LHAV and might be a signature radiological sign of LHAV. The usefulness of VTSS as a diagnostic sign of LHAV was then evaluated in another bicenter validating cohort of 1,346 patients. As shown in Table 3, in total, VTSS was detected in 230 (16.1%) patients, of whom 208 (90.4%) were confirmed to have LHAV. There were 22 patients who were positive for VTSS but not LHAV, and 8 patients had LHVA but did not exhibit VTSS. Thus, the overall sensitivity, specificity, positive and negative predictive value of VTSS for predicting LHAV were 96.3%, 98.3%, 90.4% and 99.4%, respectively.

We further demonstrated if the slice thickness of scan could affect the recognition of VTSS. We analyzed all the 108 patients whose VTSS was found by CT scan in the validating cohort. The CT thickness was set to 1.25 mm, 2.5 mm, 5 mm and 7 mm, respectively and VTSS was recognized in all patients at all thicknesses (data not shown).

## Discussion

This study is the first systematic analysis, to our knowledge, focusing on the AA courses through FLV. Our results demonstrated that the manifestation of AA in FLV occurred in approximately 15% of the patients receiving TACE. Anatomically, these AAs heterogeneously consisted of at least six different arteries, namely replaced LHA, accessory LHA, CHA, accessory LGA, LIPA and the common trunk of accessory LGA and LIPA, more importantly, 90% of the AAs seen in FLV were either replaced or accessory LHA originating from the LGA, The above finding strongly indicated that VTSS is a signature radiological sign of LHAV.

The VTSS we presented in this study provided an easy, specific and cost effective method for preoperative awareness of LHAV, which might substantially reduce the risk of complications caused by the ignorance of variant arteries. By recognizing VTSS, radiologists might take into account the high possibility of LHAV and be prepared when dealing with unexpected situations caused by aberrant LHA during TACE procedures.

Another important finding of our study is that the FLV seems to be the only portal for the aberrant arteries to either enter or leave the left hepatic lobe, considering the incidence of observing an aberrant artery in this area could be as high as 15%. FLV appears to be a critical anatomical structure that should receive more attention not only from radiologists but also from surgeons. We highly suggest radiologists and surgeons carefully observe the FLV on contrast-enhanced CT or MRI before all procedures, and when VTSS is found, essential preparations should be considered and arranged. In patients who will receive left-hepatic related procedures, further examination such as CTA might be required to obtain a more detailed profile of the hepatic arteries.

Our study had several limitations. First, we used both CT and MRI image for various diagnostic purposes to determine the presence of VTSS, and this lack of consistency might impact the precise evaluation of the whole system. Second, VTSS is generally a subjective judgment in this present study that could be influenced by a doctor’s experience and multiple other factors, and a quantitative definition of VTSS in the future will provide a solution to this issue. Third, about 10% of patients with VTSS do not exhibit LHAV, although we had some preliminary experiences such as the non-LHAV arteries were usually thinner and shorter in VTSS, our current method was not able to distinguish them from those that do exhibit LHAV.

In conclusion, in this study, we described an interesting radiological sign—the VTSS. By analysing the anatomical composition of the AA composing VTSS in a large-scale series of 2,275 patients, we established and validated a strong association between VTSS and LHAV. As a signature radiological sign, VTSS showed a sensitivity of 96.3% and a specificity of 98.3% for the diagnosis of LHAV. Finally, it has to be pointed out that despite being useful, VTSS could only be complementary tool but not a replacement of CTA, with particular usefulness in cases when CTA is not available.

## Materials and Methods

The study protocol was reviewed and approved by the institutional review board (IRB) of the 302 Hospital, Beijing, China and the general hospital of PLA, Beijing, China. Written informed consent was waived by the IRB of both hospitals because this is an observational respective study and all of the data used were acquired for diagnostic or therapeutic purposes and were generated before the study was designed.

### Patients

Between August 2012 and August 2013, 2,275 patients with hepatocellular carcinoma (HCC) on whom TACE was performed at the 302 hospital, Beijing, China were enrolled as the training cohort for analysing the incidence and anatomical compositions of the AAs seen in VTSS. To validate the usefulness of VTSS as a potential diagnostic sign of LHAV, 962 and 543 patients with HCC that received TACE between September 2013 and March 2014 at the 302 hospital and the general hospital of PLA, respectively, were enrolled to generate a bicenter validating cohort. All of the patients in the training and validating cohort had complete analysable hepatic arteriography data and received a contrast enhanced scan by either CT or MRI before TACE. Data from the training cohort were analysed by two independent radiologists (Y.L. and X.W.) and were confirmed by a third when their judgments were in conflict (Q.Y.). Data from the validating cohort were analysed blindly by another two independent radiologists (Z.W. and J.D.).

### Hepatic Arteriography and TACE Procedure

TACE was performed as previously described^7^. After local anaesthesia, the femoral artery was accessed using the standard Seldinger technique. A 5-F RH catheter was then conducted, through which arteriography of the celiac trunk, superior mesenteric artery (SMA) and hepatic arteries were successively performed to collect the overview of the hepatic arterial blood supply and the location of HCC. Upon the identification of HCC and its tumor feeding artery, a 3-F microcatheter was coaxially inserted through the 5-F RH catheter into the tumor feeding artery for superselective embolization with an emulsion of lipiodol and cytotoxic drugs.

### CT and MRI Scan

Contrast-enhanced CT and/or MRI scans of the abdomen were performed in all patients for various diagnostic purposes. CT scan was performed by a GE Light-speed VCT 64 slice scanner (GE Healthcare, Milwaukee, WI, USA). MRI scan was performed by a GE Signa HDx 1.5T scanner or a GE Signa HDx 3.0T scanner (both from GE Healthcare, Milwaukee, WI, USA). The scans followed the protocol for standard contrast-enhanced abdominal CT or MRI examination as previously described^8^,^9^,^10^. The slice thickness was 1.25 mm, 2.5 mm, 5 mm, and 7 mm, respectively, for determining the effects of slice thickness on the recognition of VTSS and was 5 mm for both CT and MRI in other analysis.

### Image Analysis and the Definition of VTSS and LHAV

VTSS was defined as the recognition of an enhanced vessel coursing through the FLV in the arterial phase of an axial CT or MRI scan at the level of the 10^th^ or 11^th^ thoracic vertebrae. Typical manifestations of VTSS were depicted in Fig. 1 and supplementary Figs 1–5. For patients with VTSS, the anatomical property of the aberrant artery in VTSS was further analysed. HAV was evaluated based on the hepatic arteriography data and was classified as type I to type X according to Michel’s classification^5^. LHAV was defined as patients with a replaced or accessory LHA or a common hepatic artery(CHA) originating from the LGA, i.e., Michel’s type II, IV, V, VII, VIII and X.

## Additional Information

**How to cite this article**: Rong, G. *et al.* The "Vessel through Strait" Sign is a Signature Radiological Sign for the Diagnosis of Left Hepatic Artery Variation. *Sci. Rep.* **6**, 23922; doi: 10.1038/srep23922 (2016).

## Supplementary Material

Supplementary Information

## Figures and Tables

**Table 1:** Anatomical compositions of the aberrant arteries seen in VTSS.

Artery in VTSS	Cases	Percentage	Blood Direction
Replaced LHA	247	69.1%	Entering liver
Accessory LHA	64	17.9%	Entering liver
Variant CHA	8	2.2%	Entering liver
Accessory LGA	25	7.0%	Leaving liver
LIPA	3	0.8%	Leaving liver
Accessory LGA & LIPA	10	3.0%	Leaving liver
Total	357	100%	Entering liver (89%) Leaving liver (11%)

LHA, left hepatic artery; CHA, common hepatic artery; LGA, left gastric artery; LIPA, left inferior phrenic artery.

**Table 2:** The globe profile of HAV in 2,275 patients and the incidence of VTSS according to Michel’s classification of HAV.

Type	Cases (n, %)	VTSS positive (n, %)	Description
Left Hepatic Artery Variation (n = 324, 14.2%)			
II	193 (8.5%)	192(94.4%)	Replaced LHA from LGA
IV	50 (2.2%)	49 (98%)	Replaced RHA and LHA
V	58 (2.5%)	55 (94.8%)	Accessory LHA
VII	0 (0%)	0 (0%)	Accessory RHA and LHA
VIII	9 (0.4%)	9 (100%)	Replaced RHA and Accessory LHA
	6 (0.3%)	6 (100%)	Replaced LHA and Accessory RHA
X	8 (0.4%)	8 (100%)	CHA from LGA
Normal Anatomy (n=1,566, 68.8%)			
I	1,566 (68.8%)	34 (2.2%)	Normal
Right Hepatic Artery Variation and others (n = 385, 17.0%)			
III	123 (5.4%)	2 (1.6%)	Replaced RHA from SMA
VI	13 (0.6%)	0 (0%)	Accessory RHA
IX	80 (3.5%)	0 (0%)	CHA from SMA
NC	169 (7.4%)	2 (1.14%)	Not classifiable
Total	2,275(100%)	357(15.7%)	

LHA, left hepatic artery; CHA, common hepatic artery; LGA, left gastric artery; RHA, right hepatic artery; SMA, superior mesenteric artery; NC, not classifiable.

**Table 3:** The sensitivity and specificity of VTSS for the diagnosis of LHVA.

Center I (n = 962)		
	Real LHAV (n = 147)	Real Non-LHAV(n = 815)
VTSS positive	144	12
VTSS negative	3	803
Center II (n = 543)		
	Real LHAV (n = 69)	Real Non-LHAV(n = 474)
VTSS positive	64	10
VTSS negative	5	464
Overall sensitivity	208/216 × 100% = 96.3%	
Overall specificity	1267/1289 × 100% = 98.3%	
Overall PPV	208/230 × 100% = 90.4%	
Overall NPV	1267/1275 × 100% = 99.4%	

PPV, positive predict value; NPV, negative predict value.

## References

[b1] Lee K. H. *et al.* Transcatheter arterial chemoembolization for hepatocellular carcinoma: anatomic and hemodynamic considerations in the hepatic artery and portal vein. , Radiographics. 22, 1077–1091 (2002). 12235337 10.1148/radiographics.22.5.g02se191077

[b2] Winter T., Nghiem H. V., Freeny P. C., Hommeyer S. C. & Mack L. A. Hepatic arterial anatomy: demonstration of normal supply and vascular variants with three-dimensional CT angiography, Radiographics. 15, 771–780 (1995). 7569128 10.1148/radiographics.15.4.7569128

[b3] Song S. Y. *et al.* Celiac axis and common hepatic artery variations in 5002 patients: systematic analysis with spiral CT and DSA. Radiology. 255, 278–288 (2010). 20308464 10.1148/radiol.09090389

[b4] Ishigami K. *et al.* Accessory left gastric artery from left hepatic artery shown on MDCT and conventional angiography: correlation with CT hepatic arteriography. Am. J. Roentgenol. 187, 1002–1009 (2006). 16985149 10.2214/AJR.05.1114

[b5] Michels N. A. Newer anatomy of the liver and its variant blood supply and collateral circulation, Am. J. Surg. 112, 337–347 (1966). 5917302 10.1016/0002-9610(66)90201-7

[b6] Hiatt J. R., Gabbay J. & Busuttil R. W. Surgical anatomy of the hepatic arteries in 1000 cases, Ann. Surg. 220, 50–52 (1994). 8024358 10.1097/00000658-199407000-00008 PMC1234286

[b7] DeCecco C. N. *et al.* Anatomic variations of the hepatic arteries in 250 patients studied with 64-row CT angiography. Eur. Radiol. 19, 2765–2770 (2009). 19471940 10.1007/s00330-009-1458-7

[b8] Zhou L. *et al.* Enhanced Therapeutic Efficacy of Combined Use of Sorafenib and Transcatheter Arterial Chemoembolization for Treatment of Advanced Hepatocellular Carcinoma, Jpn. J. Clin. Oncol. 44, 711–717 (2014). 24855686 10.1093/jjco/hyu068

[b9] Wang C. P. *et al.* Hepatic Angiomyolipoma Mimicking Hepatocellular Carcinoma: Magnetic Resonance Imaging and Clinical Pathological Characteristics in 9 Cases, Medicine. 93, e194 (2014). 25526436 10.1097/MD.0000000000000194 PMC4603092

[b10] Wang H. *et al.* CT Manifestations of Spontaneous Right Posterior Portal Vein-IVC Shunts in Patients with Liver Cirrhosis. Chin. J. Med. Imag. 11, 353–354(2003).

