# Supplementary material for: The “Vessel through Strait” Sign is a Signature Radiological Sign for the Diagnosis of Left Hepatic Artery Variation
Source: Sci Rep. 2016 Apr 4;6:23922. doi: 10.1038/srep23922 (PMC4819219; doi:10.1038/srep23922)
Supplement: Supplementary Information [file srep23922-s1.pdf]

Supplementary Figures

Manuscript Title：The "Vessel through Strait" Sign is a Signature Radiological Sign for the Diagnosis of Left Hepatic Artery Variation

Author List: Guanghua Rong, Zhijun Wang, Ximing Wang, Qiang Yu, Lin Zhou3,Huaming Wang, Junhua Zhang, Jinghui Dong, Wei Ma, Weimin An, Hui Ren, Zhen Zeng Yinying Lu, Yongwu Li.

Supplementary Figure 1

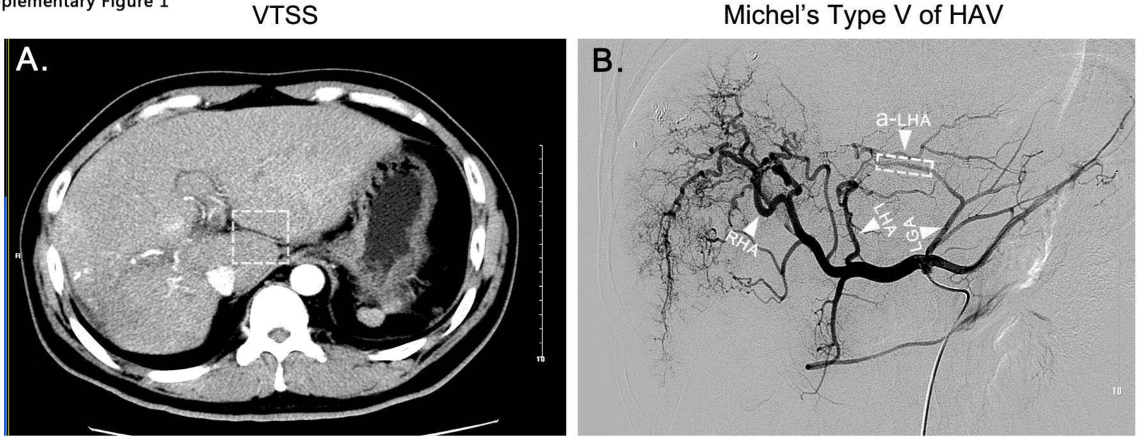

Patient No.0775

Supplementary Figure 2

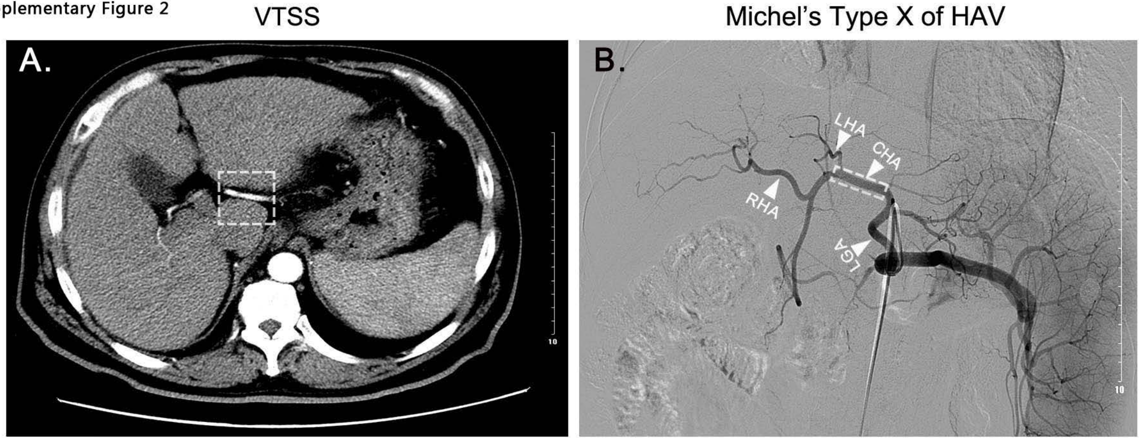

Patient No.0008

Supplementary Figure 3

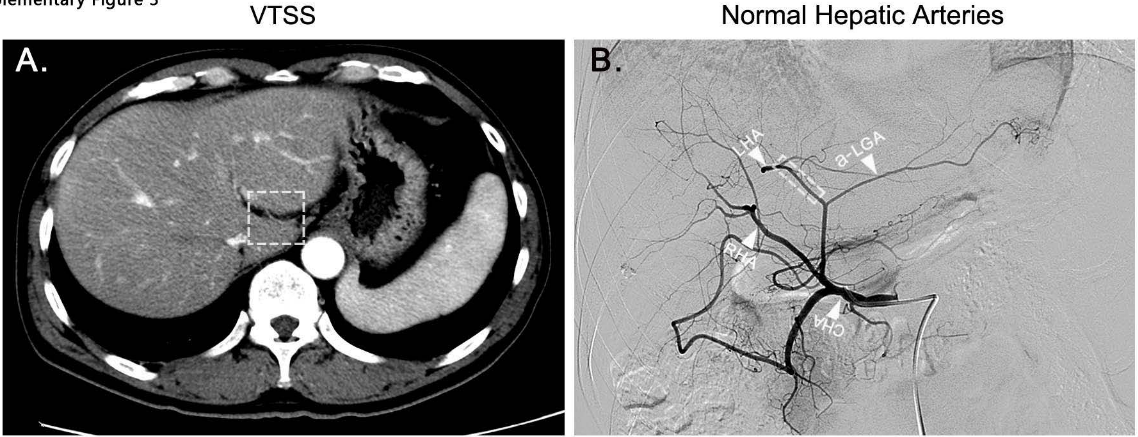

Patient No.0675

Supplementary Figure 4

VTSS | Normal Hepatic Arteries

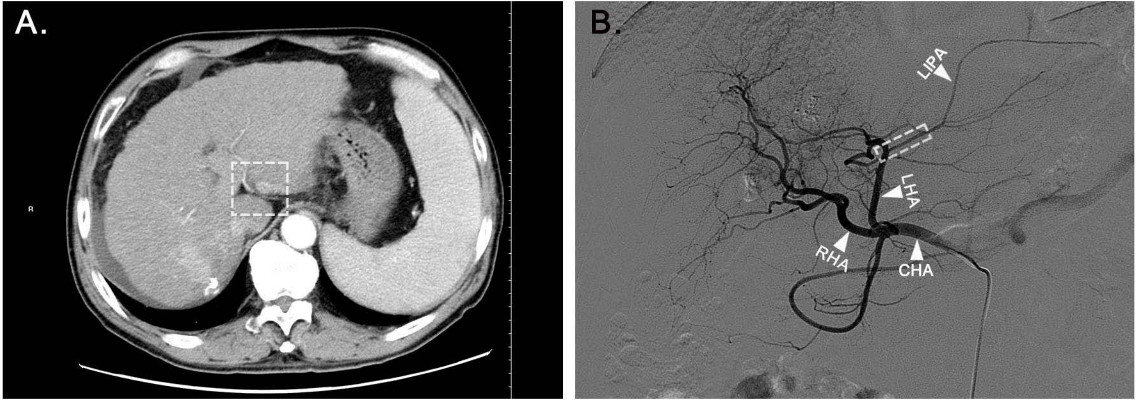

Patient No.1450

Supplementary Figure 5

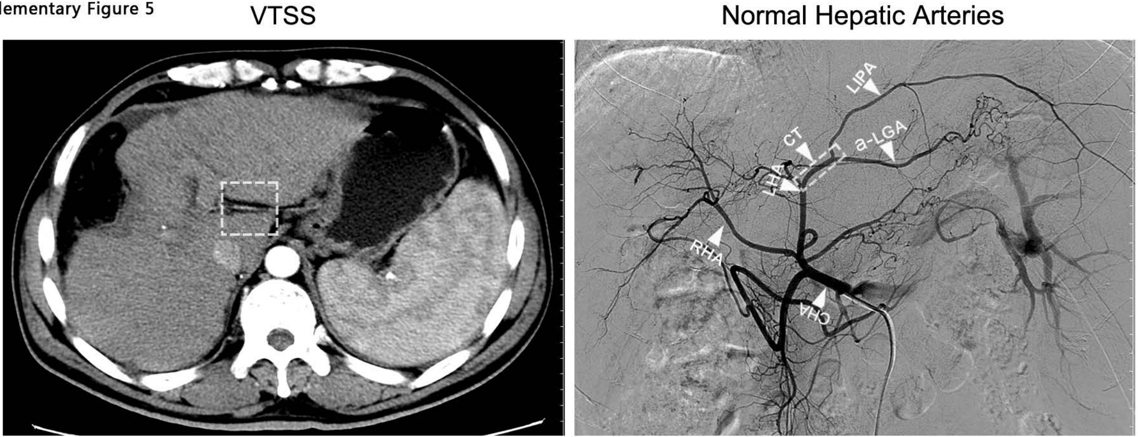

Patient No.0209

Supplementary Figure Legends

Supplementary Figure 1. (**A.**) A typical VTSS (white dashed square) formed by an accessory LHA entering liver through FLV was seen in the arterial phase of a contrast-enhanced CT scan. (**B.**) The patient was confirmed to have an accessory LHA along with a conventional LHA (Michel's type V HAV) by DSHA, the corresponding part of the accessory LHA forming VTSS was highlighted by a white dashed rectangle. a-LHA, accessory left hepatic artery.

Supplementary Figure 2. (**A.**) A typical VTSS (white dashed square) formed by an aberrant CHA entering liver through FLV was seen in the arterial phase of a contrast-enhanced CT scan. (**B.**) The patient was confirmed to have an aberrant CHA originating from LGA (Michel's type X HAV) by DSHA, the corresponding part of the aberrant CHA forming VTSS was highlighted by a white dashed rectangle.

Supplementary Figure 3. (**A.**) A typical VTSS (white dashed square) formed by an accessory LGA leaving the liver through FLV was seen in the arterial phase of a contrast-enhanced CT scan. (**B.**) The patient was confirmed to have normal hepatic artery with an accessory LGA originating from LHA. The corresponding part of the accessory LGA forming VTSS was highlighted by a white dashed rectangle. a-LGA, accessory left gastric artery.

Supplementary Figure 4. (**A.**) A typical VTSS (white dashed square) formed by a LIPA leaving the liver through FLV was seen in the arterial phase of a contrast-enhanced CT scan. (**B.**) The patient was confirmed to have normal hepatic artery with a LIPA originating from LHA. The corresponding part of the LIPA

forming VTSS was highlighted by a white dashed rectangle. LIPA, left inferior phrenic artery.

Supplementary Figure 5. (**A.**) A typical VTSS (white dashed square) formed by a common trunk of LIPA and a-LGA leaving the liver through FLV was seen in the arterial phase of a contrast-enhanced CT scan. (**B.**) The patient was confirmed to have normal hepatic artery with both LIPA and a-LGA originating from a common trunk (CT) arising from LHA. The VTSS was formed by their common trunk (white dashed rectangle). CT, common trunk; LIPA, left inferior phrenic artery; a-LGA, accessory left gastric artery.
